# The complete chloroplast genome sequence of *Eriocaulon nepalense* (Eriocaulaceae)

**DOI:** 10.1080/23802359.2019.1688704

**Published:** 2019-11-21

**Authors:** RongYan Deng, Jian He, ZhiXiang Zhang

**Affiliations:** a School of Ecology and Nature Conservation, Beijing Forestry University, Beijing, P. R. China;; b College of Forestry, Guangxi University, Nanning, P. R. China

**Keywords:** *Eriocaulon*, chloroplast genome, *Eriocaulaceae*, phylogeny

## Abstract

The complete chloroplast (cp) genome sequence of *Eriocaulon nepalense* was sequenced and assembled in this study. The cp genome of *E. nepalense* is 150947 bp in length, composed of a pair of 26451 bp inverted repeat regions (IRs), separated by a large single-copy region (LSC) of 81064 bp, and a small single-copy region (SSC) of 16981 bp. The cp genome contained 114 unique genes, including 80 protein-coding genes, 30 tRNA genes, and 4 ribosomal RNA genes. The phylogenetic position of *E. nepalense* based on the cp genome data is closer to *E. decemflorum* than *E. buergerianum*.

*Eriocaulon* Linn., under the family Eriocaulaceae, consists of 470 species in the world (WCSP 2019). These species are mainly distributed in tropical and subtropical regions, with a concentration in Asia (Ma et al. 2000). *Eriocaulon nepalense* is a common wetland plant in the Himalayan region (Zhang 1999). It is differed from other *Eriocaulon* species for having broad leaves, gray-black inflorescence, and usually apex concave at the female flower petal (Ma et al. 2000). However, none chloroplast genome resource is available so far for this species. In this study, the complete chloroplast (cp) genome of *E. nepalense* is reported and its phylogenetic position is presented.

The sample was collected from Shangsi County, Guangxi Province, China (2157′26.76″N, 107°54′25.02″ E, Voucher No. SS002, deposited at the Herbarium of Beijing forestry University). Total genomic DNA was extracted from the fresh leaves of *E. nepalense* individual using CTAB method (Doyle and Doyle 1987). Then, an Illumina HiSeq 4000 platform at Novogene (http://www.novogene.com, China) was applied to perform 2 × 150 bp pair-end sequencing. Clean reads were mapped to published chloroplast genome of *Eriocaulon* as references (Darshetkar et al. 2019) using Map function of Geneious R11 (Kearse et al. 2012). Filtered reads were then used for *de novo* assembly with Geneious R11. Gaps were bridged using Fine Tuning function of Geneious R11. The cp sequence was annotated using Plann (Huang and Cronk 2015).

The complete cp genome sequence of *E. nepalense* is 150947 bp in length. It comprises a pair of IRs (26451 bp) separated by the LSC (81064 bp) and SSC (16981 bp) regions. The cp genome harbors 114 functional genes, including 80 protein-coding (PCGs), 30 transfer RNA (tRNA) genes, and 4 rRNA genes. Among them, 10 protein-coding and 6 tRNA genes have introns. The average GC content of cp genomes is 35.8%.

Some available complete cp genome sequences of *Eriocaulon* and closed genera were downloaded from GenBank. Bayesian inference analyses (Ronquist and Huelsenbeck 2003) were conducted for phylogeny reconstruction (Figure 1). The result showed that *E. nepalense* is sister to *E. decemflorum*.

**Figure 1.**
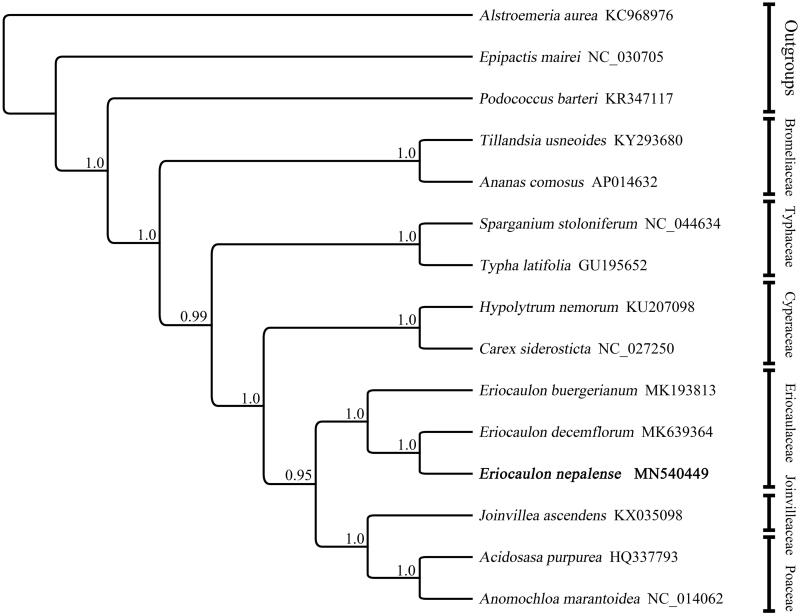
Bayesian phylogram of newly assembled genome, as well as 11 species of Poales inferred from the complete plastome sequences. PP (posterior probabilities) values for Bayesian analysis are shown at each node. NCBI accession number is shown after each species name.

The sequence alignment and all the settings of Bayesian analyses were the same with previous study by Darshetkar et al. (2019). The phylogenetic framework of *Eriocaulon,* as well as its related genera were consistent with all the previous studies (Darshetkar et al. 2019; Han et al. 2019).

## References

[CIT0001] Darshetkar AM, Datar MN, Tamhankar S, Li P, Choudhary RK 2019 Understanding evolution in Poales: insights from *Eriocaulaceae* plastome. PLoS One. 14(8):e0221423. 31430346 10.1371/journal.pone.0221423 PMC6701780

[CIT0002] Doyle JJ, Doyle JL 1987 A rapid DNA isolation procedure for small quantities of fresh leaf tissue. Phytochem Bull. 19:11–15.

[CIT0003] Han BQ, Tan GG, Hu ZY, Wang YQ, Liu Y, Zhou RC, Zhou QJ 2019 The complete chloroplast genome of *Eriocaulon sexangulare* (*Eriocaulaceae*). Mitochondr DNA B. 4(1):666–667.

[CIT0004] Huang DI, Cronk Q 2015 Plann: a command-line application for annotating plastome sequences. Appl Plant Sci. 3(8):1500026. 10.3732/apps.1500026 PMC4542940 26312193

[CIT0005] Kearse M, Moir R, Wilson A, Stones-Havas S, Cheung M, Sturrock S, Buxton S, Cooper A, Markowitz S, Duran C, et al. 2012 Geneious Basic: an integrated and extendable desktop software platform for the organization and analysis of sequence data. Bioinformatics. 28(12):1647–1649. 22543367 10.1093/bioinformatics/bts199 PMC3371832

[CIT0006] Ma WL, Zhang ZX, Thomas S 2000 *Eriocaulon nepalense* Prescott ex Bongard In: Wu ZY, Raven P, editors. Flora of China. Vol. 24 Beijing, China: Science Press and St. Louis: Missouri Botanical Garden Press; p. 11–12.

[CIT0007] Ronquist F, Huelsenbeck JP 2003 MrBayes 3: Bayesian phylogenetic inference under mixed models. Bioinformatics. 19(12):1572–1574. 12912839 10.1093/bioinformatics/btg180

[CIT0008] WCSP 2019 WCSP: world checklist of selected plant families. London: Royal Botanic Gardens; [accessed 2019 Oct 6]. https://wcsp.science.kew.org/.

[CIT0009] Zhang ZX 1999 Monographie der Gattung *Eriocaulon* in Ostasien. Dissertationes Botanicae Band 313. J. Cramer Berlin and Stuttart

